# Emotional and Psychological Experiences of Nursing students caring for Dying Patients: A phenomenology study at Mulago National Hospital, Uganda

**DOI:** 10.21203/rs.3.rs-4323878/v1

**Published:** 2024-05-08

**Authors:** Asha K. Nabirye, Ian G. Munabi, Aloysius G. Mubuuke, Sarah Kiguli

**Affiliations:** Makerere University College of Health Sciences; Makerere University College of Health Sciences; Makerere University College of Health Sciences; Makerere University College of Health Sciences

**Keywords:** Emotional experience, Psychological experiences, Nursing students, Caring for Dying Patients, Mulago National Hospital

## Abstract

**Introduction:**

Caring for dying patients is associated with psychological trauma, strong emotions and enormous stress for nursing staff and nursing students who are relied on by patients and relatives in such difficult situations. Although nurses have an ability of self-control and calm approach towards death, there are still some emotions they need to “work through”. Research studies have documented limited exposure of nursing students to end-of-life care and inadequate understanding of the psychological and emotional experiences they encounter during clinical placements. This study explored the psychological and emotional experiences of Ugandan student nurses on caring for the dying patients at Mulago national referral hospital during clinical placement.

**Methods:**

A qualitative phenomenological study was conducted among fifteen undergraduate nursing students of Makerere University in clinical placement at Mulago hospital. An In-depth interview guide was used to gather data on nursing students’ emotional and psychological experiences and coping mechanisms. Data was audio recorded, verbatim transcribed and thematically analyzed using Atlas. ti version 6 software.

**Results:**

The nursing students emotional and psychological experiences when caring for dying patients were emerged into two themes; (1) Psychological and emotional reactions, (2) Coping mechanisms. The sub themes were; anger, anxiety and depression which is triggered by a combination of issues of pressure from relatives, failure to save the dying patient, thoughts of wasted efforts to reverse the dying process, limited resources, limited technical and emotional support. The students cope by seeking help from peers, engagement in problem solving, distancing from patients, spirituality and engaging in personal stress reducing activities.

**Conclusion:**

Insights from this study provide educators with a snapshot of student encounters, emotions, and coping strategies when facing dying patients and their families. Nursing students experience various negative emotional and psychological stressors triggered by a combination of issues that need to be addressed during care of dying patients. However, they devise different coping mechanisms to continue with provision of necessary end of life care as the clinical placement contributes to their learning, experience and builds confidence among student nurses.

## Background

Death is an inexorable emotionally and psychologically stressful experience for both dying patients and those around the[1]. Caring for dying patients is a challenging task for the nursing students and staff who are resourceful in care provision during these difficult situations[2]. Although nurses have an ability of self-control and calm approach towards death, they are faced with strong emotions [3] that they need to “work through” because their professional requirement is demanding[4] and could lead to occupational burnout[5]. Nurses experience stress triggered by observation of dying patients[1] which affects their professional[6] and private life and therefore may develop preference for settings without terminal patients due to the perceived inadequacy and fear of ineffectiveness[7]. Research studies have documented compassion fatigue, related to prolonged exposure to suffering, work stress, excessive pathogenic guilt, greater emotional exhaustion, distancing, and depersonalization in nurses with greater fear and avoidance attitudes towards death[8] as emotional effects among nurses caring for dying patients.

Nursing students are likely to suffer unpleasant emotions when their patients pass away and face difficulties due to illogical cognition, somatization [9]. This leads to frustration and tension that could have a detrimental effect on their career decisions because they frequently have a lasting effect of feeling unprepared and compromise the quality of care provided[10], particularly in highly specialized settings[11]. Various styles of coping with stress can be noticed among nurses depending on job seniority and place of employment[1], however, coping by making meaning and narrative during distressing life events has been shown to minimize depressive symptoms and lead to more goal-concordant choices about end-of-life care. A study in UK showed nurses cope with stress by development of expertise in end-of-life care giving by investment in the nurse patient relationship, management of emotional labour and development of emotional intelligence[12].

Although Nursing students are subjected to clinical practice during clinical educations, research has deduced that insufficient attention is devoted to death and dying-related subjects in nursing education and many nursing students remain unprepared to manage death and care of dying patients[5]. Understanding the emotional and psychological aspects of caring for dying patients is crucial to allow nurses manifest mental maturity to required extent, inspiration of nursing students to value life and develop individual professional identities, help with patients’ understanding of meaningfulness of life, cope with distresses[13] and reduction of patient and family emotional distress. Nurses need to have effective coping strategies for attitude change towards death to address the dying patients’ end of life care demands such as spiritual comfort, peace, acceptance and deep connection with others.

Despite the available literature on end of life care experiences, especially from developed countries, the psychological and emotional experiences of nursing students caring for dying patients has not exhaustively been studied to draw adequate attention in developing countries. The primary research question of this study was ‘What are the psychological and emotional experiences of nursing students caring for dying patients during ward round rotations at Mulago hospital?’ This study explored the psychological and emotional experiences of Ugandan student nurses on caring for the dying patients at Mulago national referral hospital during clinical placement.

## Materials and methods

### Study design

The study employed a qualitative phenomenological approach. In-depth interviews were conducted at the department of nursing with undergraduate nursing students of Makerere University in the clinical years of study that had been for clinical placement at Mulago hospital which is their practicum site.

### Study site

The study was carried out at Mulago hospital which is a national referral hospital founded in 1913 and a teaching facility of Makerere University Collage of health Sciences. This site was chosen because, it is Uganda’s national and teaching hospital with 1790 beds with specialized services admitting over 140,000 patients and with 600,000 out patients’ attendance annually. Therefore has the greatest number of critically ill patients that would serve the purpose of the study [14].

### Participant selection

The study included undergraduate nursing students at Makerere University who had been for clinical placement at Mulago hospital, provided care for the dying patient in last 6months prior to data collection and consented to take part in the study.

Purposive sampling method was used for selection of students for the in-depth interviews until data saturation point was attained at 15 participants. The actual study population was the undergraduate nursing students at Makerere University in year three and four, which are their clinical years of study, who had been for clinical placement at Mulago hospital, cared for dying patients in the last six months prior to data collection and consented to the study. The study did not include nursing students who were not physically available at the time of data collection and those that who were ill to participate in the study.

### Data Collection

Two research assistants who were nurses with experience in gathering qualitative data, conducted in-depth interviews between 14th and 24th June of 2023 from the undergraduate nursing students of Makerere university. A total of fifteen in depth interviews were conducted by the use of an interview guide as the primary investigator was noting the nonverbal responses. The interviews were conducted in English language, lasted between 25 and 60 minutes, audio recorded and transcriptions extracted out of the recordings for thematic analysis in Atlas.ti version 6.

### Data management and analysis

#### Data Analysis

After each interview the audio recordings were transcribed verbatim and exported to the Atlas.ti version 6 software for analysis using an Inductive thematic approach. Each transcript was opened in the Atlast.ti 6 program, and each statement was read and coded line by line.

The student nurses were interviewed independently at their convenient time and place at the hospital campus and their perceptions considered to ensure validity of the results. To ensure conformability, each interview was checked throughout data collection process and the recorded interviews were verbatim transcribed. During analysis the primary investigator first read through the transcriptions for familiarity. Then the transcribed interviews were inductively coded through a combination of open, descriptive and structural coding systems. The statements from interview transcripts were broken into discrete extracts which were described using the descriptive codes, while some statements were grouped together for a structural flow of the responses. A code book was developed to further examine the coding with some codes being combined and others being modified to create themes. After identification of main and minor themes, and the table of topics was exported into Word for additional data interpretation. Peer debriefing was done through discussing the data collection process and findings with colleagues to provide alternative perspectives which was useful to enhance the validity of the findings. The primary investigator observed and took notes during data collection by the research assistants to further validate the findings.

## Results

There were fifteen participants in this study of whom nine were males and six were females, three students and twelve students were from third and fourth year of their study.

From the analysis of the data, the statements of nursing students were grouped under into two themes main themes twelve sub themes which were drawn from twenty three codes. The themes that emerged were (1) Psychological and emotional reactions, (2) Coping mechanisms during the experience.

**Table:** 

Themes	Sub themes	Codes
**1.Psychological and emotional reactions**	1.1 Anger	Code1.1.1: Feeling expressed during process of caring for dying patient :Anger due to failure to save patient
	1.2 Anxiety	Code1.2.1: Anxiety due to limited experience, fear to be blamed by relatives, have no answers for relatives Code 1.2.2 first time shock, blunt feeling, pressured to do something by relatives, Code1.2.3: fearful: scary, panicking, confused, helpless, struggle alone
	1.3 Depression	Code1.3.1: demoralized, felt wasted efforts, low energy Code1.3.2:saddened due to failure to save patient, emotionally drained, saddened because there are no resources to save patients, productive shame(guilt feelings if you don’t care)
**2.Coping mechanisms during care of dying patients**	2.1 Ask others to help or assist you.	Code3.1.1: learn from others, team work, get encouragement from peers, peer support, Code3.1.2: maintain technically supportive relationships: sharing experiences with fellow medical students, interacting with fellow students, Engage with colleagues, utilize health professional’s support, Avoid being alone, Communication with supervisor regularly on phone,
	2.2 Engage in problem solving.	Code3.2.1: attend to debriefs from seniors, avoid productivity shame, attempt to rescue patient, clinically manage, don’t neglect patient Code4.1.2b: continue care for patient for relatives’ sake, continue to offer care, use available resources to help
	2.3 Distance yourself from the source of stress.	Code 3.3.1: change environment to rid of emotions Code3.3.2: prefer to avoid physical presence, avoid the death situation
	2.4Spiritual support	Code3.4.1: praying, relied on personal faith & beliefs, encouraged relatives to pray, positive talk with patients to reassure them, comfort them Code3.4.2: Maintain emotional composure: encouraged oneself to emotionally support patient, not feeling so nervous, accept death is a reality
	2.5 Engage in stress-reducing activities	Code3.5.1: engage in hobbies to reduce hospital stress, Socialize

Each of the above themes and sub-themes are explained below along with key representative quotations:

### Theme 1: Psychological and Emotional reactions

This theme relates to the conscious mental reactions of nursing students in response to involvement in care of dying patients at Mulago hospital.

#### Anger

Anger feelings during physical process of caring for dying patients was triggered by various factors such as un necessary pressure, over expectation from relatives and failure to save the dying patient.

‘…relatives expect much from me yet I don’t actually give them much….. they had much expectation even when they were told that the patient would eventually die… (Student Nurse Participant 3)

‘….I still had that pain in me like I felt that I failed…. because I had been the one caring for the patient and now observing the person dying in front of me really angered and scared me…’ (Student Nurse Participant 1).

#### Anxiety

Anxiety and worry were triggered by the limited experience, fear to be blamed by relatives, lack of answers for relatives, limited knowledge and skill to operate some medical equipment or perform some medical procedures and limited support from other health professionals. First time encounters were shocking to students as they felt pressured to do something by relatives. Students further described their feelings as fearful, scary, helpless, panicked, blunt and confused about how to approach the physical process of care.

…. I felt I was going to be blamed for what had happened… (Student Nurse Participant 1)

‘…….I had that anxiety of performing some procedure, because I was scared, what if this one is going to be done to kill this patient?’ (Student nurse participant 3).

‘…. I felt so anxious to go and tell the caretakers that your mother is dying….’ (Student Nurse Participant 7).

‘…I was just hearing about central lines, but I had actually never cared for a patient with a central line…it was my first encounter…….I was fearing to go towards and approach the patient …”. (Student Nurse Participant 10)”.

#### Depression

They were saddened due to failure to save patient, emotionally drained and at times saddened because there are no resources to save patients and yet felt guilty if they didn’t care for the patients. The participants felt demoralized after confirmation that they are caring for a dying patient, had low drive and were saddened that their efforts could not change much or reverse the dying process.

….I think, I didn’t have adequate knowledge of what should be done in that moment, what should be said, where the patient should be touched, whom should I call and this really affected my thoughts and I felt sad d…’ (Nurse participant 10)

‘…… I had a feeling that maybe I would have done something special, but the resources were not there and so I became so sad …..” (Student Nurse Participant 8)

### Theme 2: Coping mechanisms during care of dying patients

This theme pertains to the attempts and adjustments of nursing students when dealing with difficulties involved in caring for the dying patients.

#### Asking others for help or assistance

Participants acknowledged that they obtain peer support through which they learn how to manage the negative experiences of caring for dying patients. Participants mentioned that they get help from conserved technically supportive relationships which allow sharing of experiences with fellow medical staff, interactions with fellow students, and utilization of health professional’s support.

“….I kept on learning a lot and interacting with my fellow students,……we kept on strengthening each other….”(Student Nurse Participant 1).

….. after some time, I had to accept that the person is dying and I can’t reverse it…. after that I could, communicate with my friends and talk about it…’ (Student Nurse Participant 4).

#### Engage in problem solving.

Some participants cope through applying tips from attended debriefs of senior health professionals, avoid productivity shame by attempting to rescue patient or clinically manage patient. The participant also ensures continuation of care and use available resources to help the patient and relatives.

‘……I stopped being alone …so I always kept engaging myself in other conversations with my colleagues….. So, it was a kind of debrief. Debrief whenever something scary would come up….” (Student nurse participant 1)

#### Distance yourself from the source of stress

Some participants prefer to avoid physical presence with dying patient through change of environment to get rid of negative emotions.

‘…Most guys try to refrain from the situation. I don’t know why or because of their experience and their schedule… ” (Student nurse participant 4).

‘….I started feeling the panic. So now, sometimes when you panic, you might actually do wrong things, even things you’ve always been perfect at. I cope by avoiding that vicinity…. because now, if I didn’t know you, I have to disappear the fact that I don’t even know what to do, I rather avoid than maybe making things worse…. and so I avoid physical presence… ” (Student nurse participant 5).

#### Spiritual support

Some participants rely on their personal faith and beliefs such as praying for divine intervention, to ensure their personal emotional stability and also to encourage, reassure and comfort the patients and their relatives. Some participants had mastered the art of emotional composure by encouraging themselves personally to emotionally support patient, not feeling so nervous and accept death as a reality.

‘…… actually, I am a Catholic and I wished maybe if the dying patients were also Catholics, because for us, they bring the priests and then they give them their last blessing so maybe they go well…..……wwhen coping with the situation, I could go to church and pray afterwards…and then, I get things that distract me….’ (Student nurse participant 2).

#### Engaging in stress-reducing activities

Participants expressed their engagement in various hobbies and social net working to reduce hospital stress.

‘…. when coping with the stress, If I could go home, I could enjoy my hobbies, I enjoy listening to music…’(Student Nurse Participant 3)

## Discussion

This study was set out to explore the psychological and emotional experiences of nursing students caring for the dying patients and the main findings were that students experience various psychological and emotional reactions, and devise coping mechanisms to deal with difficulties encountered in caring for dying patients.

### Psychological and emotional reactions

In this study nursing students expressed anger, anxiety, fear and depression which are likely to be triggered more often during care of dying patients as nurses frequently interact with the patients. Similar to a study done in Togo[15],results from this study highlighted pressure and failure to save the dying patient could have triggered anger, anxiety and emotional distress during physical process of care of dying patients. Studies have shown that interactions with dying patients stimulate negative feelings of anxiety, stress and burnout and perceptions [16].

In this study, depression could also have been triggered by the thoughts of wasted efforts to reverse the dying process, limited resources, and limited technical and emotional support to student nurses during care of dying patients.

However, a study in Poland highlights empathy, sadness and helplessness as common nurses’ emotions in care of dying patients, regardless of the nurses’ length of service and the place of work[1]. Similar to a study in UK[17],nursing students in this study were intimidated by the process of communication to the relatives hence the anxiety they expressed during the care of dying patients. The breaking of bad news and interactions with relatives are sources of emotional stress for nurses caring for dying patients that needs to be given attention during the education curriculum of nurses.

Management of psychological and emotional reactions is important to ensure provision of quality care among dying patients. Some studies [18],have shown that in such difficult situations nursing students’ fear of losing control and not being able to support patient and relatives in an appropriate and sensitive way generates anxiety, terror, and emotional distress.

These findings are similar to study done in Asia 2013 that showed nurses experienced great mental and physiological strain regarding caring of dying patients[19].Therefore its important ensure adequateknowledge levels among nursing students regarding care of dying patients to boost their confidence and minimize negative emotional reactions. This could be done by ensuring provision of adequate and relevant theoretical training combined with emotional intelligence components with allowance for students to express their fears before the clinical practicum. Studies have documented Education of emotional intelligence components can improve the efficiency of nursing care services and professional competence due to decreased stress[20] and increased feelings of security among students [21].

Also peer coaching needs to be encouraged among senior students and junior students, students and health professionals on ward during placement to improve on emotional intelligence abilities of the nursing students[22].

Relaxation techniques, training in behavioral techniques, stress management workshops and training in therapeutic skills are also effective stress management techniques and should be encouraged by hospital administrators regularly[23].

### Coping strategies with the negative experience

Results in this study showed the students use peer support in fostering learning as a coping strategy to manage difficult situations during clinical placements. These results are similar to a study done in another University of Cape coast in Ghana that showed students seek support from peers and create healthy relationships and avoid clinical environment as coping mechanisms[24]. Peer support is considered crucial for nurses because it’s a resource and an opportunity to share experiences, feelings and hardships with colleagues facing death of patients[25]. Peer support, is one of the most common coping strategies applied by nurses involved in the care of dying children.

This study showed students tend to keep distance from the dying patient when dealing with emotional stress. Distancing has been a documented strategy used by students to manage negative experiences in clinical settings[25]. This concurs with findings from various studies that showed health professionals avoid dying patients[15], and students cope with clinical stressors by avoidance[26].There is need to prioritize care for the dying patients and ensuring open communication with patient and relatives to support dignified, honest and respectful death for the dying patient.

Engagement in problem solving by ensuring continuity of care has been embraced by the nursing students in this study. This concurs with findings from Spain[27] and USA[28] that showed that when dealing with negative experiences in clinical settings, the coping strategy most frequently used by students was problem-solving. Similar to a study in India, debrief sessions in this study were helpful in sharpening the student nurses’ problem solving skills. The debriefs are important and should be encouraged to relate with nurses’ emotions, experiences and responses to death.

Similar to Indian study, spiritual beliefs were a form of personal coping strategy used by nursing students. In this study spirituality seemed to be a hobby for nurses to manage emotional stress of carrying for dying patient[29]. Students should therefore be equipped with coping mechanisms for dealing with negative experiences and hospital care stressors during care of patients.

### Strength and limitations of the study

This present study was the first of its kind among nursing students thus provided a baseline data.

A limitation of this study pertains to the self-report methodology employed for data collection. The responses provided by participants might have been influenced by social desirability bias, as well as their inclination to portray themselves in a positive manner. This potential influence could have implications for the accuracy and depth of the insights conveyed, potentially resulting in a less exhaustive comprehension of the perceptions and experiences under investigation

## Conclusion

Nursing students experience anger, anxiety and depression which is triggered by a combination of issues such as; pressure from relatives, failure to save the dying patient, thoughts of wasted efforts to reverse the dying process, limited resources, limited technical and emotional support from senior health professionals and supervisors during care of dying patients. However, they devise different coping mechanisms during the process of caring for dying patients that includes, obtaining support from peers, avoidance /distancing from patients, engagement in problem solving, turn to spirituality or engage in stress reducing activities.

Therefore, its important ensure adequate knowledge levels among nursing students regarding care of dying patients to boost their confidence and minimize negative emotional reactions.

This could be done by ensuring provision of adequate and relevant theoretical training combined with emotional intelligence components with allowance for students to express their fears before the clinical practicum.

## Recommendations

Proper preparation, orientation and training of health professionals and student nurses on the care of dying patients should be done by respective heads of departments regularly to foster professional development and patient care.

Appropriate Information sharing and open communication should be encouraged among health professionals and care givers/ family/relatives of dying patients and nursing students to ease communication and reduce pressure from relatives during care.

## Future research

There is need for further investigations on the factors that could influence the emotional and psychological reactions and coping mechanisms among nursing students in the Ugandan context.

## Data Availability

Data sources are available on request. The request can be sent to the corresponding author at ashanabirye1@gmail.com
